# Physiological and unappreciated roles of CaMKII in the heart

**DOI:** 10.1007/s00395-018-0688-8

**Published:** 2018-06-15

**Authors:** Jan Beckendorf, Maarten M. G. van den Hoogenhof, Johannes Backs

**Affiliations:** 1 Department for Molecular Cardiology and Epigenetics, University Hospital Heidelberg, Im Neuenheimer Feld 669, 69120 Heidelberg, Germany; 2 Department for Cardiology, Angiology and Pneumology, University Hospital Heidelberg, Heidelberg, Germany; 3 DZHK (German Centre for Cardiovascular Research), Partner Site Heidelberg/Mannheim, Heidelberg, Germany

**Keywords:** Calcium, Calmodulin, CaMKII, Cardiomyocyte, Inflammation, Apoptosis

## Abstract

In the cardiomyocyte, CaMKII has been identified as a nodal influencer of excitation–contraction and also excitation–transcription coupling. Its activity can be regulated in response to changes in intracellular calcium content as well as after several post-translational modifications. Some of the effects mediated by CaMKII may be considered adaptive, while effects of sustained CaMKII activity may turn into the opposite and are detrimental to cardiac integrity and function. As such, CaMKII has long been noted as a promising target for pharmacological inhibition, but the ubiquitous nature of CaMKII has made it difficult to target CaMKII specifically where it is detrimental. In this review, we provide a brief overview of the physiological and pathophysiological properties of CaMKII signaling, but we focus on the physiological and adaptive functions of CaMKII. Furthermore, special consideration is given to the emerging role of CaMKII as a mediator of inflammatory processes in the heart.

## Introduction

Heart failure is one of the most prevalent diagnoses upon hospital admission and, despite all therapeutic progress over the last decade, is still associated with a high rate of morbidity and mortality [54, 77]. In the diseased myocardium, CaMKII plays central roles in processes such as maladaptive remodeling [1, 3, 44, 45, 48, 50, 72, 115, 117], arrhythmogenesis [63], interstitial fibrosis [3, 45] and apoptosis [21, 22, 103, 112]. As such, CaMKII is a promising target for pharmacological inhibition and the development of inhibitory compounds is racing ahead [73]. Two compensatory mechanisms during heart failure are (a) an excessive production of catecholamines and (b) the activation of the renin–angiotensin–aldosterone system. For each of these mechanisms, CaMKII has been shown to play an integral role in conveying the following (mal)adaptive processes, leading to cardiac remodeling and heart failure [18, 30, 113]. However, while there is vast knowledge of the role of CaMKII in cardiac disease, the role of CaMKII in physiological processes is less well studied. This review aims at highlighting the sparse insights into the physiological role of CaMKII signaling in the heart and also its role in some underappreciated inflammatory processes in the heart.

## CaMKII structure and activity

Calcium(Ca^2+^)/calmodulin(CaM)-dependent kinases (CaMK) are serine/threonine (Ser/Thr)-specific phosphokinases. They respond to changes in intracellular [Ca^2+^], which is the major second messenger inside the cardiomyocyte and indispensable for the coupling of membrane excitation with myofibril contraction, also termed excitation–contraction coupling (ECC) [55]. As free calcium ions are quickly removed from the cytosol during diastole, they can be bound by the Ca^2+^-sensor calmodulin [14] to allow the exertion of functions that last longer than just one depolarization, resp. one systole, especially on gene transcription or epigenetic regulation, often termed excitation–transcription coupling (ETC) [8]. An increase of [Ca^2+^] inside the cardiomyocyte leads to the activation (and potentially overactivation) of calcium-dependent signaling. As a result, overall CaMKII activity is upregulated ~ 3-fold in human heart failure [42], and the expression rate of CaMKIIδ was shown to be increased ~ 2-fold [33].

The structure of the functional CaMKII enzyme is dodecameric, taking the form of two stacked hexameric rings [13]. Each monomer consists of an N-terminal catalytic domain and a C-terminal association domain. In between, an autoregulatory domain, which also includes the Ca^2+^/CaM binding site, regulates the activation status through Ca^2+^/CaM-binding and also by autophosphorylation [35]. Ca^2+^/CaM-dependent activation of CaMKII is dependent on total [Ca^2+^]_*i*_ in a dose-dependent manner, but also on Ca^2+^ spark frequency, amplitude and duration, as well as the previous activation state [15]. When inactive, the catalytic domain is sterically blocked by the regulatory domain, a mode also referred to as the autoinhibitory state. CaMKII is activated upon Ca^2+^/CaM binding to the CaM-binding site of the regulatory domain, leading to a conformational change, which exposes the kinase substrate and adenosine triphosphate (ATP) binding sites of the catalytic domain [81]. When one monomer enters the active state, the regulatory domains of neighboring CaMKII monomers become available for autophosphorylation at Thr287 (in CaMKIIδ, the exact numbering changes slightly between different CaMKII isoforms), furthering CaMKII activation and also blocking re-association of the catalytic domain with the autoinhibitory domain [35, 47], maintaining kinase activity even after dissociation of the Ca^2+^/CaM complex. Autophosphorylation of Thr287 leads to another interesting effect called CaM trapping, in which CaM binding affinity is increased 1000-fold, keeping the Ca^2+^/CaM complex in place and thus sustaining CaMKII activity under conditions of low [Ca^2+^]_*i*_ [61]. Further research unveiled other mechanisms of CaMKII activation via post-translational modifications (PTM) of the regulatory domain that are Ca^2+^/CaM independent, such as oxidation of the Met281/282 residues by reactive oxygen species (ROS) [18] and S-Nitrosylation of Cys290 through a nitric oxide (NO)-dependent pathway [19], and O-GlcNAcylation at Ser279 during hyperglycemia [20]. However, these mechanisms still need the initial activation of CaMKII via the canonical Ca^2+^/CaM binding. Eventually, CaMKII can be inactivated via dephosphorylation of Thr287 by protein phosphatase 2A (PP2A) or protein phosphatase 1 (PP1) [96]. Another phosphatase-independent mechanism for negative regulation of CaMKII activity exists via autophosphorylation of Thr305/306, preventing CaM from binding to the regulatory domain again once it dissociated from its binding site (CaM-capping) [78].

## CaMKII genes and splice variants

The group of calcium/calmodulin-dependent kinases consists of three classes: CaMKI, CaMKII and CaMKIV. CaMKII is further distinguished by its four isoforms α, β, γ, δ—each isoform being encoded by a separate gene [98]. The expression rates of CaMKII isoforms differ between tissue types. CaMKIIα and β are predominantly expressed in neuronal tissue, while the CaMKIIδ and γ isoforms can be found in cells of almost any differentiation [34]. CaMKIIδ and γ are the main isoforms found in cardiac tissue, with the δ isoform outweighing the γ isoform ~ 2.5-fold [3].

**Fig. 1:**
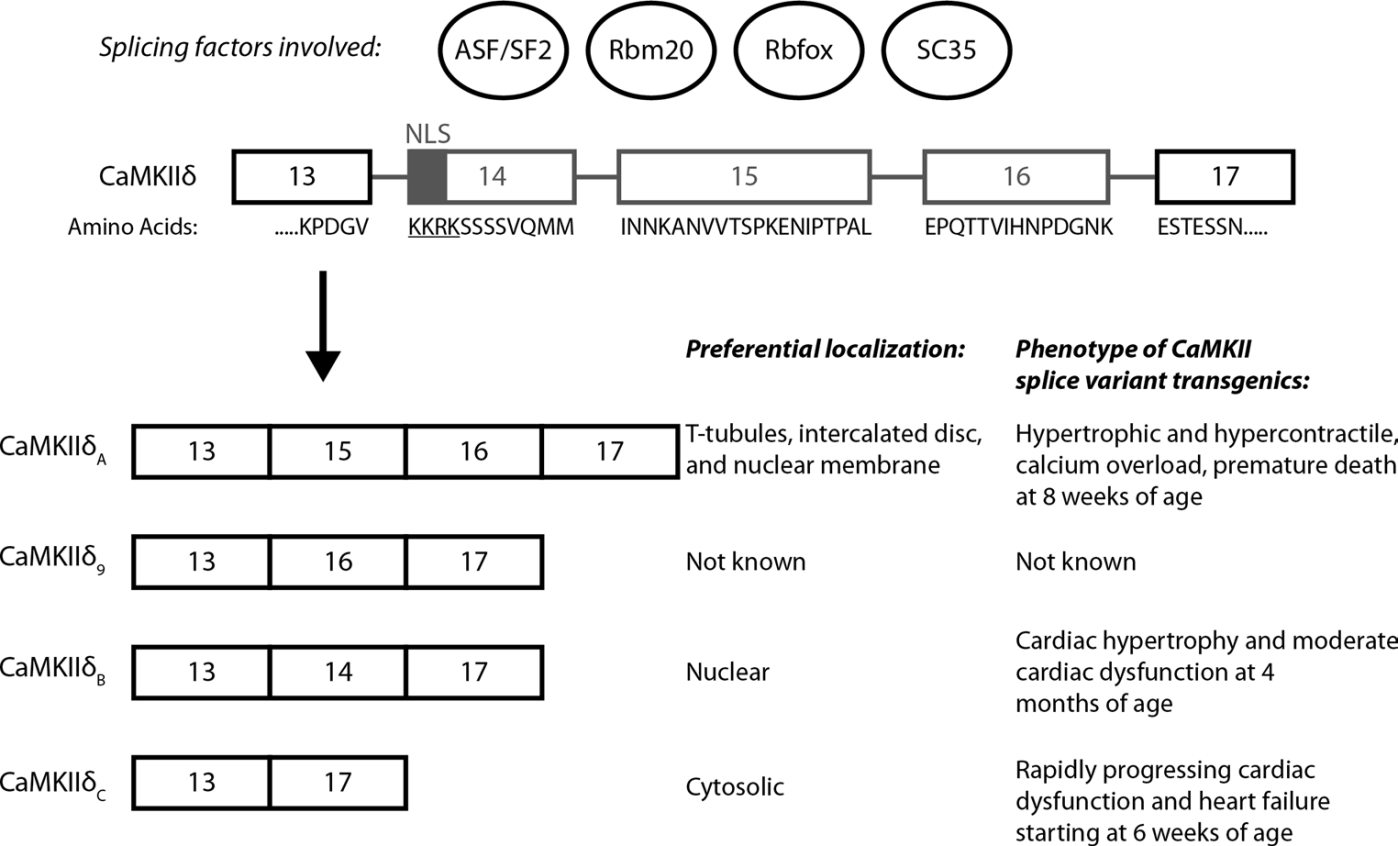
Alternative splicing of CaMKIIδ. Alternative exons (exons 14–16) in the pre-mRNA are depicted in gray. Differential alternative splicing gives rise to the different CaMKIIδ isoforms, which have different preferential cellular localizations and potentially different functions. Exon 14 contains a nuclear localization signal (NLS) and the serine (Ser322) adjacent to the NLS can posttranslationally be modified to affect nuclear localization

All CaMKII genes are subjected to alternative splicing, but CaMKIIδ splicing is most well studied in the heart. Alternative splicing of CaMKIIδ results in at least 11 different splice variants, among which the δ_A_, δ_B_, δ_C_ and δ_9_ are most seen in the heart (Fig. 1) [24]. The δ_A_ splice variant preferentially localizes to t-tubules, sarcolemmal and nuclear membranes, and is implicated in the formation of maladaptive cardiac hypertrophy after catecholaminergic stimulation [48, 111]. CaMKIIδ_B_ uniquely contains a nuclear localization sequence (NLS) and thus predominantly localizes to the nucleus, while CaMKIIδ_C_ mainly localizes to the cytosol [95]. At the moment, very little is known about CaMKIIδ_9_, but as it resembles CaMKIIδ_A_ the most, it may function in a similar manner. Three splice variants of CaMKIIγ have been found in the heart [88], but in contrast to the CaMKIIδ splice variants, the respective function of each CaMKIIγ splice variant in the heart is unknown. Each completely assembled dodecamer is constructed of different isoforms and splice variants. It is thought that the relative predominance of a certain splice variant in the heteromultimer might confer the target specificity for the respective cell compartment of the entire enzyme [62]. There is evidence that the differential compartmentalization of the splice variants also reflects differences in function, as the δ_B_ variant may predominantly regulate transcription and the δ_C_ variant may rather influence excitation–contraction coupling [114]. The different functions and relative importance of the splice isoforms of CaMKIIδ are, however, far from clear. For instance, it has been shown that δ_C_ is also able to block the nuclear import of histone deacetylase 4 (HDAC4), thereby possibly affecting gene expression as efficient as the δ_B_ variant [4, 116]. Systematic analyses of these different splice variants in different stages of cardiac development and disease are therefore awaited with great interest.

CaMKIIδ splicing is regulated by at least two different splicing factors, ASF/SF2 and Rbm20 [26, 111]. Members of the Rbfox protein family and SC35 have also been implicated in CaMKIIδ splicing, but their in vivo relevance is less clear [29]. Interestingly, during development CaMKIIδ switches from the CaMKIIδ_A_ splice variant, to the CaMKIIδ_B_ and CaMKIIδ_C_ variants, and loss of either ASF/SF2 or Rbm20 leads to persistent expression of fetal CaMKIIδ_A_. It has been hypothesized that CaMKIIδ_A_ is necessary for enhanced L-type calcium current in neonatal cardiomyocytes, as they rely on L-type calcium current instead of calcium-induced calcium release (CICR) for contraction [27, 111]. While it is not yet known how CaMKIIδ_A_ enhances L-type calcium current, this hypothesis is in line with the observed increased calcium transients in ASF/SF2 knockout (KO) mice and CaMKIIδ_A_-TG mice [111]. Interestingly, this effect seems to be gender dependent, as only male ASF/SF2 KO and CaMKIIδ_A_-TG mice were affected. Very recently, van den Hoogenhof et al. found that Rbm20 KO mice have an intracellular Ca^2+^ overload, which leads to spontaneous Ca^2+^ releases from the SR [101]. It seems likely that this underlies the increased risk of arrhythmias in RBM20 mutation carriers. Interestingly, this Ca^2+^ overload was due to increased L-type Ca^2+^ current density, and as loss of Rbm20 also induces a shift to the fetal CaMKIIδ_A_ isoform, this is completely in line with the hypothesized function of CaMKIIδ_A_.

CaMKIIδ_B_ is involved in remodeling via the epigenetic regulator HDAC4 during pathological pressure overload [4, 116]. However, it was also suggested that CaMKIIδ_B_ might mediate cardioprotective effects, as it strongly suppresses cardiomyocyte apoptosis after doxorubicin treatment and during oxidative stress [51] [74]. Nevertheless, CaMKIIδ_B_ transgenic mice develop hypertrophy and moderate cardiac dysfunction at 4 months of age [115]. Transgenic overexpression of CaMKIIδ_C_ in mice, on the other hand, results in a rapid progression of heart failure and premature death [117], and Sossalla et al. demonstrated the role of CaMKIIδ_C_ in diastolic dysfunction and arrhythmogenesis [93]. However, in contrast to these previous findings, the collaborative work of our laboratory with Wolfgang Linke pointed to a reduction in passive stiffness of cardiomyocytes after phosphorylation of the sarcomeric structure protein titin by CaMKIIδ_C_, improving diastolic filling, an effect that may be partially beneficial in diastolic dysfunction [28]. The latter finding warrants further investigations to explore its functional relevance in in vivo situations including diastolic dysfunction. However, functional redundancy among the different CaMKII genes and perhaps with other related kinases including protein kinase D complicate such studies because they require breeding of different mouse models.

## Physiological and adaptive functions of CaMKII

As CaMKII is a ubiquitously expressed and multifunctional kinase, its function and importance have been studied in a multitude of tissues. Outside the heart, CaMKII is has been shown to be critically involved in vital processes like memory formation through long-term potentiation [2], hepatic glucose production and insulin signaling [69, 70], vascular smooth muscle cell function [99], cell cycle progression and fertility [5, 39], as well as the immune system [10]. In the heart, the role of CaMKII under conditions of pathological cardiac stress has been studied extensively. However, relatively little is known about the role of CaMKII in the non-diseased heart after physiological stimuli, as well as its possible adaptive roles in the diseased heart. The newly generated conditional KO models [3, 5] of the two ubiquitously expressed CaMKII genes δ and γ might provide a toolbox that allows to identify unknown essential CaMKII functions.

CaMKII is recognized as an instrument of the cell for the fine-tuning of its intracellular calcium content, especially concerning the ECC in myocytes. During the plateau phase of the action potential, calcium shifts into the cell through L-type calcium channels (LTCC), which leads to a relatively low increase of subsarcolemmal calcium in the dyadic cleft between the sarcolemma of the T-tubules and the sarcoplasmic reticulum. There, each LTCC is juxtaposed by a cluster of ryanodine receptors (RyR2). The initial calcium influx is followed by an amplifying mechanism called calcium-induced calcium release (CICR), during which even more calcium is quickly released from the sarcoplasmic reticulum through the ryanodine receptor, boosting [Ca^2+^]_*i*_. Thereby, the binding of free cytosolic calcium with troponin C is made possible, which then leads to the conformational change of the tropomyosin/actin complex and enables myosin binding, ultimately leading to myofilament contraction [92]. During diastole, free calcium is rapidly removed from the cytosol, either by transport into the extracellular space through the sodium/calcium exchanger (NCX) or by reuptake into the SR via the SR-Ca^2+^-ATPase (SERCA).

These processes can be regulated by CaMKII: CaMKII can, for example, phosphorylate several subunits of the LTCC, thereby increasing Ca^2+^-dependent facilitation of the LTCC [36, 43]. In addition, CaMKII phosphorylates the sarcoplasmic reticulum (SR) membrane protein-complex phospholamban (PLN) at Thr17 [87], leading to increased calcium reuptake from the cytosol into the SR via SERCA2a [59]. Lastly, the ryanodin receptor 2 (RyR2), which is located in the sarcoplasmic reticulum membrane, is phosphorylated by CaMKII at Ser2809 [107] and more importantly Ser2814 [102, 104], leading to reduced SR [Ca^2+^] through increased SR calcium leak into the cytosol. The details of CaMKII and its role in ECC and ETC, however, are beyond the scope of this review and both have previously been reviewed in depth by many investigators including Lars Maier [55] and Donald Bers [8], respectively.

CaMKII is not only pivotal for calcium handling in ECC and ETC, but is also required for the increase in heart rate (HR) after β-adrenergic stimulation, also known as the fight or flight response [109]. Sinoatrial node (SAN) cells rely on an inward ‘pacemaker’ current through HCN4, leading to faster action potential generation, but HCN4 KO mice retain their ability to increase HR after β-adrenergic stimulation. Wu and colleagues showed that activation of CaMKII in SAN cells enhances SR Ca^2+^ filling and release, and increases the diastolic depolarization rate. This leads to faster action potential generation, independent of HCN4 current. Interestingly, CaMKII inhibition only affects HR after β-adrenergic stimulation, and not at baseline. It must be noted that this effect did not depend on a single CaMKII in PLB or RyR2, but rather that the concerted action on multiple phosphorylation targets decreases SR Ca^2+^ content below a certain threshold which seems to be required for the fight or flight response [110].

A recent study showed that CaMKII is centrally involved in the adaptive contractile response after aerobic training, and therefore indispensable for the adequate response of the heart to a physiological stimulus [12]. Mechanistically, this effect was shown to depend on increasing levels of insulin-like growth factor 1 (IGF-1) after exercise, which leads to activation of the nitric oxide (NO) synthase 1 (NOS-1) through the PI3K/Akt pathway. This, in turn, leads to activation of CaMKII, putatively through the NO-dependent S-nitrosylation of Cys290, resulting in the enhancement of calcium cycling through SERCA2a and the desirable effects of increased inotropy and lusitropy. Interestingly, blockade of CaMKII with the inhibitory peptide AC3-I abolished the effects on contractility and relaxation, but not the cardiomyocyte hypertrophy.

Along those lines, our laboratory, using CaMKIIγ/CaMKIIδ double knockout (DKO) mice, showed that pathological and physiological cardiac hypertrophy in mice was not primarily CaMKII dependent, but rather attributable to the calcineurin (CnA)–NFAT axis, while CaMKII was responsible for maladaptive effects, i.e., systolic and diastolic dysfunction [44]. A similar observation that hypertrophy was independent of CaMKII while maladaptive remodeling did require CaMKII was made by the group of Joan Heller Brown [50]. At baseline, CaMKIIγ/CaMKIIδ DKO mice exhibit a slight increase in contractile force, but even though PLN-Thr17 and RyR2-Ser2814 were markedly hypophosphorylated, no changes in cellular Ca^2+^ handling could be detected [44]. While this suggests that CaMKII is dispensable for normal cardiac function, CaMKII is also involved in the adaptive response after physiological stress. Upon exercise, CaMKII expression in control mice was unaltered, but activity was decreased by 30%. Even though control mice had a hypertrophic response, as indicated by increased heart weight/body weight (HW/BW) ratios and cardiomyocyte hypertrophy, this did not affect cardiac function. In CaMKIIγ/CaMKIIδ DKO mice this response was exaggerated, and the CnA target gene RCAN1–4 was excessively upregulated, but cardiac function was also not affected. However, decreased CaMKII activity decreases phosphorylation of the autoinhibitory Ser411 phosphorylation site of CnA, suggesting that CaMKII is necessary to inhibit overactivation of calcineurin.

Conversely, Ole Kemi and co-workers previously showed increased cardiac contractility and Ca^2+^ cycling after aerobic interval training in adult mice, and inhibition of CaMKII using the autocamtide-2 related inhibitory peptide II (AIP) abolished these effects [41]. These animals also did not show an increase of overall CaMKII expression, but in this case CaMKII activity, as assessed by P-Thr287-CaMKII and P-Thr17-PLN, was increased. In human skeletal muscle, P-Thr287-CaMKII is increased as early as 5 min after the start of the exercise, and activity is increased after 40 min [80]. Endurance training of human skeletal muscle also increases P-Thr287-CaMKII and activity, but here P-Thr17-PLN was unaltered [79]. Currently, there is no satisfactory answer to these seemingly contradictory results, but in these studies CaMKII activity has been measured in different and indirect assays, and exercise regimens were different, which could explain the discrepancies.

Another beneficial function of CaMKII is that recovery from acidosis depends on acute CaMKII activation. Acidosis, the lowering of pH, which can be of clinical significance during myocardial infarction and cardiac ischemia, decreases contractile performance and alters intracellular calcium handling [68]. On the electrophysiological level, acidosis increases extrusion of H^+^ from the cardiomyocyte by the Na^+^/H^+^ exchanger, which increases intracellular [Na^+^]. This, in turn, increases diastolic [Ca^2+^]_*i*_ through the reverse mode of NCX. In cardiomyocytes, this activates CaMKII, which can then phosphorylate PLN to increase Ca^2+^ re-uptake by SERCA2a, ultimately leading to increased SR Ca^2+^ content and increased Ca^2+^ transients [60]. The increase in Ca^2+^ transients is pivotal in overcoming the decreased contractility during acidosis, and CaMKII activation has proven to be necessary for this coping mechanism, both in vitro and in vivo [65, 68]. However, acute activation of CaMKII also has adverse effects; for example, ethanol and doxorubicin can acutely activate CaMKII, which ultimately leads to an increased SR Ca^2+^ leak that seems to be pro-arrhythmic [64, 82]. Ethanol and doxorubicin both increase ROS production, which consequently can activate CaMKII by oxidation. Activated CaMKII is known to promote diastolic SR Ca^2+^ leak, for example by hyperphosphorylation of RyR2, which increases the open probability of the channel. Ultimately, this can repress Ca^2+^ transients and contractility and serve as a basis for arrhythmogenic effects. It must be noted that CaMKII and protein kinase A (PKA) share a number of phosphorylation targets, among which are RyR2 and PLN [23, 110]. RyR2, for example, can be phosphorylated by CaMKII at Ser2815 and by PKA at Ser2809, and both phosphorylation events increase the open probability of the RyR2 channel and are therefore pro-arrhythmic. Fisher et al. have recently shown that during hypertrophy, both CaMKII- and PKA-dependent phosphorylations of RyR2 are increased, which may induce SR Ca^2+^ leak, but during the transition from hypertrophy to heart failure, only CaMKII-dependent phosphorylation of RyR2 is increased [23]. Discussing the differential roles of CaMKII vs. PKA in the regulation of their phosphorylation targets is beyond the scope of this review, but extensive literature on this subject exists (see for example Johnston et al. [37] or Marx et al. [57]).

Nevertheless, the beneficial sides of short-term or acute activation of CaMKII need not be disregarded, and further studies are needed to unravel the relative contributions of CaMKII in the different phases of the adaptive response of heart and skeletal muscle to physiological stress. It will be interesting to identify and investigate the targets of CaMKII at different time points after physiological stimuli, to discern what mechanisms, be it calcium cycling remodeling, gene regulation, or metabolic remodeling, are most prominently affected.

## The role of CaMKII in apoptosis and necroptosis

While apoptosis (or programmed cell death) is an important physiological mechanism of well-ordered organ development, it is also one of the pathophysiological hallmarks of myocardial remodeling in heart failure where it entails detrimental effects on cardiac contractility through cell loss [40]. The role of CaMKII in apoptotic signaling in non-cardiac cancer cells was first published by Wright et al. [108] and, a few years later, Zhu et al. demonstrated that CaMKII was essential for cardiomyocyte apoptosis after beta-adrenergic overstimulation [119]. Since then, a huge body of work supports the pro-apoptotic properties of CaMKII signaling as recently reviewed by Feng and Anderson [22]. However, these experiments were mostly done using chemical or peptide-based kinase inhibition (AIP, KN-93, AC3-I), which are prone to several limitations (as discussed in [105]). In these studies, CaMKII inhibition seemed to be clearly anti-apoptotic. However, new studies indicated different roles of CaMKIIδ splice variants in apoptosis, when Peng et al. and Little et al. confirmed pro-apoptotic properties only for CaMKIIδ_C_, but unexpectedly found anti-apoptotic properties for CaMKIIδ_B_ after oxidative and doxorubicin-induced myocardial damage [51, 74]. This seemingly clear-cut picture of good and evil became muddled when our group aimed to dissect the individual roles of CaMKIIδ, CaMKIIγ and especially the CaMKIIδ_C_ and δ_B_ splice variants after experimental ischemia/reperfusion (I/R) injury [105], which is a potent driver of apoptosis [16]. Using this model, our group was unable to detect a CaMKII-dependent effect of either isoform or splice variant. In contradiction, Ling et al. demonstrated a clear increase of apoptotic cell death after I/R, which was abrogated by CaMKIIδ_C_ knockout [50]. Therefore, in I/R damage the role of CaMKII signaling must be considered unresolved.

In contrast to apoptosis, necrosis was long thought to be a passive non-ATP-dependent process of cell death, usually triggered upon, e.g., hypoxia. However, under certain circumstances, even the chaotic process of necrosis may underlie some cellular control. This regulated form of necrosis has therefore been termed necroptosis as a portmanteau of necrosis and apoptosis [46]. Necroptosis can be triggered by activation of receptor-interacting protein 3 (RIP3), a protein phosphokinase that has CaMKII as a substrate [118]. This is a unique finding, as CaMKII was previously not known to be phosphorylated by any other kinase than itself. Disruption of RIP3 or CaMKII signaling leads to a marked reduction of cell death after I/R or doxorubicin treatment. CaMKII was previously suggested to influence the opening of the mitochondrial permeability transition pore (mPTP) by increasing inner membrane mitochondrial calcium uniporter currents (*I*_MCU_), leading to depolarization of the mitochondrial inner membrane and ultimately cell death [38]. It may be speculated that through its involvement in necroptosis, CaMKII may also play a regulative role, possibly by preventing uncontrolled necrosis during cardiac injury.

## CaMKII signaling in inflammation

Recent works have placed CaMKII signaling in the middle of inflammatory processes. In immune cells, CaMKII plays a major role in the activation of T cells and the formation of T cell memory mirroring the function of CaMKII in memory formation in the brain [9, 10, 66]. Furthermore, CaMKII signaling in the immune system was found to be responsible for the pro-inflammatory cytokine production in macrophages [52, 75] and for dendritic cell function [32]. CaMKII activity is also associated with the propagation of asthmatic bronchitis through pro-inflammatory action in the airway epithelium, smooth muscle cells and mast cells and this was mostly ROS dependent [84, 86]. However, CaMKII can also be activated downstream of inflammatory stimuli such as toll-like receptor (TLR) activation [91] or interleukin-10 (IL-10) signaling [75].

In the heart, CaMKII signaling is intricately involved in the propagation of ischemic and reperfusion-associated damage to the heart muscle, thereby influencing the degree of inflammatory response and, thus, scar formation and cardiac function. The importance of CaMKII in these processes, however, has been under debate, and opposing results have been reported. Some work on this subject was done by the group of Joan Heller Brown, where in the wake of 60 min ischemia with following reperfusion for up to 24 h, cardiomyocyte-CaMKII was discovered to phosphorylate and thereby activate I kappa B kinase (IKK), leading to de-repression of nuclear factor kappa B (NF-κB), a central regulator of inflammation [49]. This effect could be diminished by inhibition of IKK, as well as genetic deletion of CaMKIIδ, leading to reduced infiltration of the ischemic muscle area by macrophages and eventually resulting in attenuated scar size and improved pump function. In a follow-up study, the respective roles of the splice variants δ_B_ and δ_C_ in the setting of injury/reperfusion (I/R) damage were examined [25]. Mice that overexpressed CaMKIIδ_C_ in a background of global CaMKIIδ deletion showed increased infarct size and systolic dysfunction. The opposite was observed in mice with isolated CaMKIIδ_B_ overexpression, where infarct size was even smaller than in the complete CaMKIIδ KO, an observation that strengthens the notion that CaMKIIδ_B_ can exert protective effects through suppression of cardiomyocyte apoptosis [51, 74]. Furthermore, it was shown that the activation of the CaMKIIδ_C_–IKK–NF-κB axis leads to increased expression of tumor necrosis factor alpha (TNFα), and inhibition of either IKK or TNFα was sufficient to reduce infarct size [25]. This pathway was previously also implied in other models of cardiac disease [89, 90]. However, it must be noted that clinical trials, examining the potential of a blockade of the mentioned pathways in the setting of myocardial infarction or heart failure so far, were disappointing, both for NF-κB inactivation through the administration of glucosteroids [11] and after treatment with the TNFα blocker etanercept [56, 71].

**Fig. 2:**
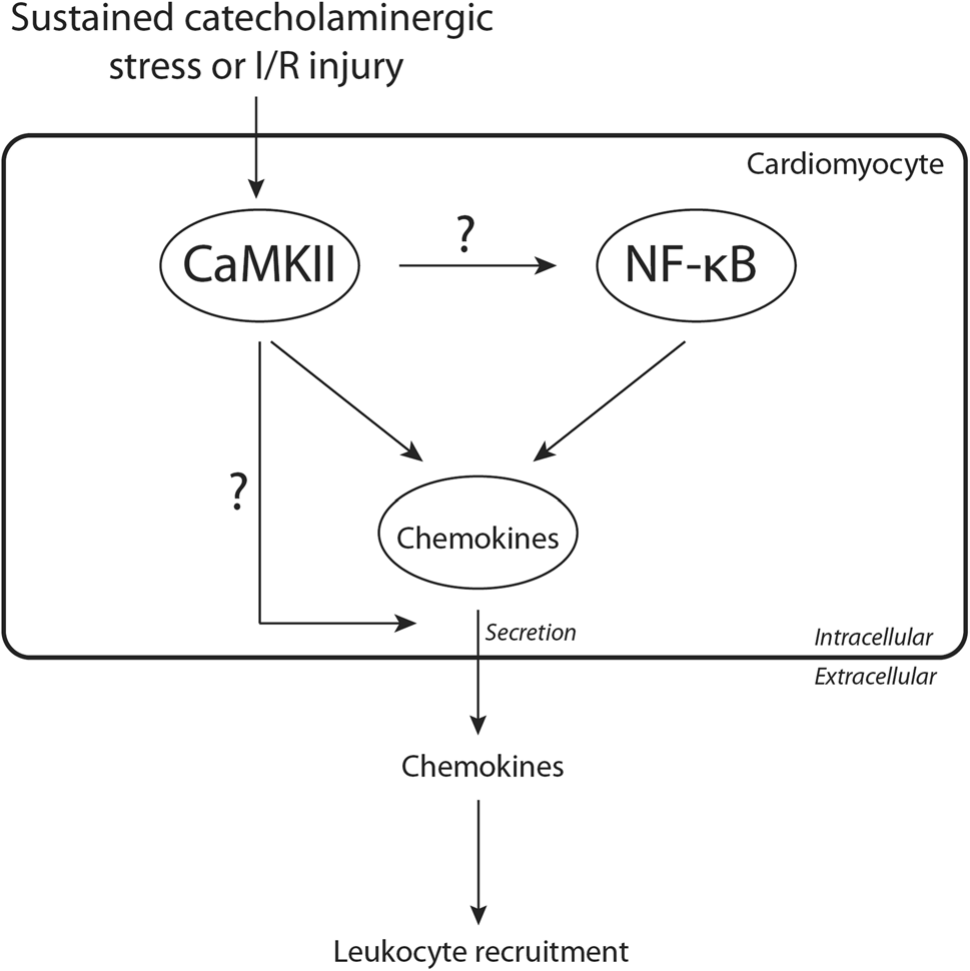
CaMKII mediates chemokine expression and secretion in/from cardiomyocytes. In response to sustained catecholaminergic stress or ischemia/reperfusion (I/R) injury, CaMKII increases expression and potentially secretion of chemokine ligands such as CCL2/3 either dependent or independent of NF-κB signaling. This figure merely illustrates a very specific role of CaMKII and for a more general overview of all CaMKII functions, we would like to direct the reader to [31, 58]

However as mentioned above, in a similar I/R study from our group, Weinreuter and co-workers did not observe a difference in infarct size or apoptosis 1 day after I/R in CaMKIIδ KO, CaMKIIγ KO and CaMKIIγ/δ DKO mice, and also after re-expression of CaMKIIδ_B_ or CaMKIIδ_C_. Only at 5 weeks after I/R, CaMKIIγ/δ DKO mice showed a reduced infarct size and improved cardiac function. This effect was associated with attenuated leukocyte infiltration and chemoattractant signaling in the hearts of CaMKIIγ/δ DKO mice, in particular in the time period from 1 to 5 days after I/R. Specifically, loss of CaMKII decreased the cardiomyocyte-intrinsic expression and secretion of the chemokines C–C motif ligand (CCL) 2 and 3, and thereby decreased scar area through diminished attraction of inflammatory cells (Fig. 2) [105]. The discrepancy between these studies may be due to the utilization of different KO strategies or the dissimilar genetic background of the animals, and future studies to investigate the potential reasons underlying the different results are needed. Since still little is known about CaMKII in the setting of chronic post-ischemic heart failure after the cessation of acute inflammatory processes, further research into the role of CaMKII in chronic post-ischemic heart failure is urgently warranted. The inflammatory processes that occur in the heart after MI have different stages, with different cell types involved, and the chemoattractant CCL2 is needed in the first stage to attract Ly-6C^high^ monocytes [97]. Ly-6C^high^ monocytes are required during the initial response, but can be detrimental if they persist too long [97]. Increased understanding of these processes, and how CaMKII is involved, might lead to new CaMKII-based therapeutic strategies that point to a specific treatment period after ischemic injury which aims to avoid infiltration of specific subsets of leukocytes into the myocardium.

CaMKII may also play an ambiguous role in angiogenesis during inflammatory conditions. Westra et al. showed that inhibition of CaMKII leads to reduced expression of hypoxia-inducible factor 1α (HIF-1α) in macrophages, thereby also decreasing the expression of vascular-endothelial growth factor (VEGF) and possibly reducing angiogenesis [106]. Additional evidence was recently provided by Banumathi et al., who showed that retinal angiogenesis is critically dependent on CaMKII, and inhibition of CaMKII with KN-93 decreased retinal angiogenesis [6]. However, after myocardial infarction, increased angiogenesis is highly desirable [85] and a potential therapeutic CaMKII inhibition might be disadvantageous regarding revascularization and collateralization of hypoxic areas.

## CaMKII in infectious disease

Of note, CaMKII signaling was discovered to be involved in the progression of Chagas’ disease by enabling heme-induced cell proliferation of the *Trypanosoma cruzi* epimastigotes [67, 94]. Chagas disease is a potentially deadly disease afflicting many Latin American regions and its incidence is currently rising due to increased population mobility and non-vectorial transmission [76, 83]. Very limited therapeutic options are available for the treatment of this disease, especially during its chronic phase [83]. Here, pharmacological inhibition of CaMKII might therefore serve as a potential anti-infective strategy. An interesting question arising from this observation is whether CaMKII signaling might also be involved in the propagation of Chagas-associated cardiomyopathy that develops in up to 30% of patients [100], considering that an effect of *T. cruzi* on cardiomyocyte calcium handling is already known [7]. This thought is especially tantalizing, as it was shown that the related *Trypanosoma brucei*, which may also confer myocardial disease, can directly induce CaMKII-mediated proarrhythmogenic SR calcium leak in cardiomyocytes [17] and an upregulation of the chemokines CCL2 and CCL3 was found in *T. cruzi*-associated cardiomyopathy [53], which, we know now, is driven by CaMKII [105]. Combining Chagas disease with CaMKII conditional KO mouse models might answer this intriguing question in the future.

## Conclusions

The role of CaMKII as a promoter of adverse cardiac remodeling, dysfunction, arrhythmia and inflammatory processes is relatively clear. However, its role in the cardiovascular physiology in response to benign stress, e.g., endurance training, is a more ambiguous one. In addition, some works even describe cardioprotective effects of CaMKII activation under certain pathological stimuli, and the essential roles of CaMKII outside the heart should not be ignored, as these poorly understood effects could have a huge impact on drug development programs and would favor a CaMKII target-specific approach over enzymatic CaMKII inhibition. Overall, the beneficial effects of acute or short-term activation should not be disregarded and, though the maladaptive effects of sustained CaMKII activation are well studied, future studies are needed to discern if CaMKII really is the foe it has been made out for or maybe has a more acute, but neglected friendly side.
